# Predictive value of cytokines combined with human neutrophil lipocalinin acute ischemic stroke-associated pneumonia

**DOI:** 10.1186/s12883-023-03488-w

**Published:** 2024-01-17

**Authors:** Mingming Zhang, Xiaoqian Shi, Bin Zhang, Yingqi Zhang, Ying Chen, Daofeng You, Hongmin Zhao, Qianqian Lu, Yanrong Ma

**Affiliations:** 1 Department of Emergency, The First Hospital of Hebei Medical Univerisity, Shijiazhuang, China; 2 Department of Clinical Laboratory, The First Hospital of Hebei Medical Univerisity, Shijiazhuang, China; 3 Department of General Practice, The First Hospital of Hebei Medical Univerisity, Shijiazhuang, China; 4 No.89 Donggang Road, Shijiazhuang, 050031 Hebei China

**Keywords:** Stroke-associated Pneumonia, interleukin-6, Human neutrophil apolipoprotein, Acuteischemic Stroke, Predictive value

## Abstract

**Objective:**

To explore the predictive value of interleukin-6 (IL-6) combined with human neutrophil lipocalin (HNL) of stroke-associated pneumonia (SAP) in patients who were diagnosed with acute ischemic stroke (AIS).

**Methods:**

108patients were divided into two groups: pneumonia group (52 cases) and non-pneumonia group (56 cases), according to whether the patients developed SAP within 7 days of admission. General information was compared between the two groups, like age, gender, history of hypertension, diabetes mellitus, cardiovascular disease, dysphagia, smoking and alcoholhistory. Clinical data were recorded and compared, including lipid profile, interleukin-6 (IL-6), homocysteine (Hcy), National Institutes of Health Stroke Scale (NIHSS) score, and HNL. Multivariate Logistic regression analysis was used to screen the risk factors of AIS-AP, and the predictive value of IL-6 and HNL alone and in combination was evaluated by receiver operating characteristic curve (ROC curve).

**Results:**

Logistic regression analysis showed that dysphagia (OR,0.018; 95% CI, 0.001 ~ 0.427; *P* = 0.013), increased NIHSS scores(OR,0.012; 95% CI, 0.000 ~ 0.434; *P* = 0.016), and high levels of IL-6 (OR,0.014; 95% CI, 0.000 ~ 0.695; *P* = 0.032)and HNL (OR,0.006; 95% CI, 0.000 ~ 0.280; *P* = 0.009) were independent risk factors for SAP with significant difference (all *P* < 0.05). According to the ROC curve analysis of IL-6, the area under the curve (AUC) was 0.881 (95% CI: 0.820 ~ 0.942), and the optimal cutoff value was 6.89 pg/mL with the sensitivity of 73.1% and specificity of 85.7%. As for the ROC curve analysis of HNL, the AUC was 0.896 (95% CI: 0.839 ~ 0.954), and the best cutoff value was 99.66ng/mL with the sensitivity of 76.9% and specificity of 89.3%. The AUC of the combination of IL-6 and HNL increased to 0.952 (95% CI: 0.914 ~ 0.989), and the sensitivity and specificity increased to 80.8% and 92.9%, respectively.

**Conclusion:**

In this research, the levels of IL-6 ≥ 6.89 pg/mL and HNL ≥ 99.66ng/mL were considered as risk factors for AIS patients complicated with SAP. The combined detection had higher predictive value for patients with SAP, which may help to identify who were in highrisk.

## Introduction

As the most common type of stroke, acuteischemic stroke (AIS) has four characteristics of high incidence, fatality, disability and recurrence [1]. It is reported that 15–25% of stroke patients die from stroke-associated pneumonia(SAP) [2], a common complication of AIS. To avoid the poor outcome of patients with AIS, researchers were to explore the potential risk factors for SAP [3], and there have been some clinical predictive models to screenpatients in high risks for SAP [4, 5]. However, these predictive models are mostly based on clinical findings, besides, the symptoms of patients are usually a typical and nonspecific. Therefore, it is important for early and accurate detection of SAP in patients with AIS.

Human neutrophil lipocalin(HNL) was first identified in human neutrophils, and now it is a novel inflammatory marker [6]. In patients with chronic obstructive pulmonary disease (COPD), the increased level of indicate that bacterial infections are the main cause of deterioration in these patients [7]. According to a study, the diagnostic value of HNL is better than that of procalcitonin (PCT) in lung infections [8]. It was reported that HNL detection was significant for the diagnosisof infected patients with the precise diagnosis [9]. Inbacterial infection, HNL level is increased, and its sensitivity and specificity are superior to that of blood neutrophil counts, and procalcitonin [10], whichmean that the finding couldbe a major insightin the managementof patients with acute infections [10].

Studies have reported that inflammatory immune response is involved in the occurrence and development of ischemic stroke, which is closely related to the severity and prognosis of stroke.In a PREDICT study, stroke-induced immunosuppression was an independent predictor of stroke-associated pneumonia, with up to 10% of those patients in the highest serum interleukin-6 (IL-6) quartile developing SAP and none in the lowest quartile developing SAP [11].Therefore, it is urgent for us to find the accurate and simple way to predict the SAP in patients with AIS.

In this study, we aimed to explore the predictive value of IL-6 and HNL alone and in combination for SAP in patients with AIS, so as to provide new ideas for the prevention and treatment of SAP.

## Patients and methods

### Objects

From June 2022 to March 2023, a total of 108 cases of elderly patients, who were admitted within 3 days of the onset of symptoms of AIS, were selected as the research objects in this retrospective study. All patients diagnosed with AIS were routinely treated with anti-platelet aggregation, plaque stabilization, blood pressure control and blood glucose control. The patients were divided into two groups: pneumonia group (52 cases) and non-pneumonia group (56 cases), according to whether the patients developed stroke-associated pneumonia (SAP) within 7 days of admission. This study was approved by the Ethics Committee of The First Hospital of Hebei Medical University (Ethics No. 20,220,537), and thepatients and their guardians provided informed consent, demonstrating their willingness to be included in the research.

### Inclusion and exclusion criteria

Inclusion criteria: (1) new-onset pneumonia within 7 days of stroke onset in non-mechanically ventilated patients [12]; (2) the brain magnetic resonance imaging (MRI), magnetic resonance angiography (MRA), and CT scan were conducted within the first 24 h in all participants.

Exclusion criteria: patients who were complicated with (1) cerebral embolism, cerebral hemorrhage after infarction, cerebral trauma; chronic cardiopulmonary insufficiency; urinary system infection; hepatic renal insufficiency; (2) hematological diseases, malignant tumors, treatment of radiotherapy and chemotherapy or biological agents; (3) systemic infectious disease or preexisting infection; (4) severe autoimmune disease.

### Clinical data

The general dataincludedage, sex, history of hypertension, diabetes, coronary heart disease, history of smoking and alcohol.The symptoms (dysphagia) and blood test results were recorded: serum total cholesterol (TC), low density lipoprotein cholesterol (LDL-C), homocysteine (Hcy). Patients’initial stroke severity was evaluatedby well-trained neurologistsdaily from admission to discharge viathe National Institutes of Health Stroke Scale (NIHSS) score [13]. The NIHSS score was performed by specially trained physicians: The total score of 42 points, mild stroke ≤ 4 points, moderate stroke: 5 ~ 15 points, severe stroke: 16 ~ 42 points, the higher the scores, the more serious the neurological function deficit.

### Laboratory examination

IL-6: After all subjects were enrolled, 5mL fasting venous blood was extracted when admission or in the morning of the next day, which was naturally coagulating at room temperature or centrifuged at 2000-4000 rpm for 20 min. About 0.5mL of the isolated serum was collected and sent for testing and IL-6 level was detected by immunofluorescence assay. The kit was purchased from Jiangxi cellgene Biotechnology Co., LTD.

HNL: After admission, 5mL of fasting venous blood was extracted at the day of admission or the next morning, and placed in a test tube without anticoagulant at room temperature for more than 2 h before serum separation, so as to facilitate the full release of HNL protein. HNL level was detected by enzymolytic immunoassay (ELISA), which was purchased from Changchun Brother Biotechnology Co., LTD.

### Statistical analyses

SPSS 20.0 software (IBM Corp, USA) was used for statistical analysis. All data were tested for normality and homogeneity of variance. The measurement data was expressed as Mean ± SD, and T test was used to compare the data between groups. Multivariate logistic regression analysis was used to analyze the influencing factors of SAP in patients with AIS. The receiver operating characteristic (ROC) curve analysis compared the predictive value of IL-6 and HNL alone or in combination for SAP. All *P*values < 0.05 were considered as significant difference.

## Results

### General data

The age in pneumonia groupranged from 53 to 90 years old, with an average of 68.65 ± 10.04 years old, including 32 males and 24 females. Innon-pneumonia group: 44 to 83 years old, mean age (66.18 ± 11.16) years old; 28 males and 24 females.There was no statistical significance in age, gender, history of hypertension, diabetesmellitus, cardiovascular disease, dysphagia, smoking and alcohol history, and basic indexes TC, LDL-C and Hcy in two groups (all *P* > 0.05). The most common site of pulmonary infection in all cases is the right middle and lower lobes of the lungs. The proportion of dysphagia and NIHSS score in pneumonia group were higher than those in non- pneumonia group (all *P* < 0.05). See Table 1.

**Table 1:** General information

	Non-pneumonia group (n = 56)	Pneumonia group (n = 52)	*t*	*P* value
Male/Female (n)	32/24	28/24	0.119	0.730
Age (year)	66.18 ± 11.16	68.65 ± 10.04	-1.213	0.228
Smoking n(%)	14(25.00)	16(30.77)	1.938	0.164
Alcohol n(%)	14(25.00)	12(23.08)	0.055	0.815
Hypertension n(%)	34(60.71)	32(61.54)	0.008	0.930
Diabetes n(%)	12(21.43)	16(30.77)	1.225	0.268
Coronary heart disease n(%)	8(14.29)	12(23.08)	1.381	0.240
Dysphagia n(%)	6(10.71)	20(38.46)	11.357	0.001
NIHSS	9.21 ± 4.47	15.27 ± 5.27	-6.414	<0.001
TC (mmol/L)	4.90 ± 0.91	4.73 ± 0.57	1.162	0.248
LDL-C (mmol/L)	3.30 ± 0.69	3.28 ± 0.39	0.113	0.910
Hcy (umol/L)	14.69 ± 6.26	14.83 ± 6.45	-0.109	0.913
Site of occluded blood vessel.			0.024	0.878
Anterior circulation.	18	16		
Posterior circulation.	38	36		

NIHSS, National Institutes of Health Stroke Scale; TC, total cholesterol; LDL-C, low density lipoprotein cholesterol; Hcy, homocysteine

### The levels of IL-6, HNL between two groups

The levels of IL-6 and HNL in pneumonia group were significantly higher than those in non- pneumonia group (all *P* < 0.05, Table 2).

**Table 2:** The levels of IL-6, HNL between two groups

	Non-pneumonia group (n = 56)	Pneumonia group (n = 52)	*T* value	*P* value
IL-6 (pg/mL)	6.50 ± 1.60	10.72 ± 3.15	-8.685	<0.001
HNL (ng/mL)	78.37 ± 20.86	122.11 ± 30.55	-8.624	<0.001

IL-6, interleukin-6; HNL, human neutrophil lipocalin

### Multivariate logistic regression for the risk factors of SAP

With the above statistically significant influencing factors as independent variables, the occurrence of SAP as dependent variables, dysphagia, increased NIHSS score, IL-6 and HNL levels as independent risk factors for SAP, as shown in Table 3.

**Table 3:** Multivariate logistic regression for the risk factors of SAP

	b	SE	Wald χ2	*P* value	OR value	OR 95%CI
Dysphagia	-4.024	1.620	6.174	0.013	0.018	0.001 ~ 0.427
NIHSS	-4.414	1.826	5.846	0.016	0.012	<0.001 ~ 0.434
IL-6	-4.302	2.010	4.584	0.032	0.014	<0.001 ~ 0.695
HNL	-5.192	2.000	6.737	0.009	0.006	<0.001 ~ 0.280
Constant term	11.906	3.507	11.524	0.001		

NIHSS, National Institutes of Health Stroke Scale; IL-6, interleukin-6; HNL, human neutrophil lipocalin

### ROC curve analysis for prediction of SAP by IL-6 and HNL alone and in combination

**Fig. 1:**
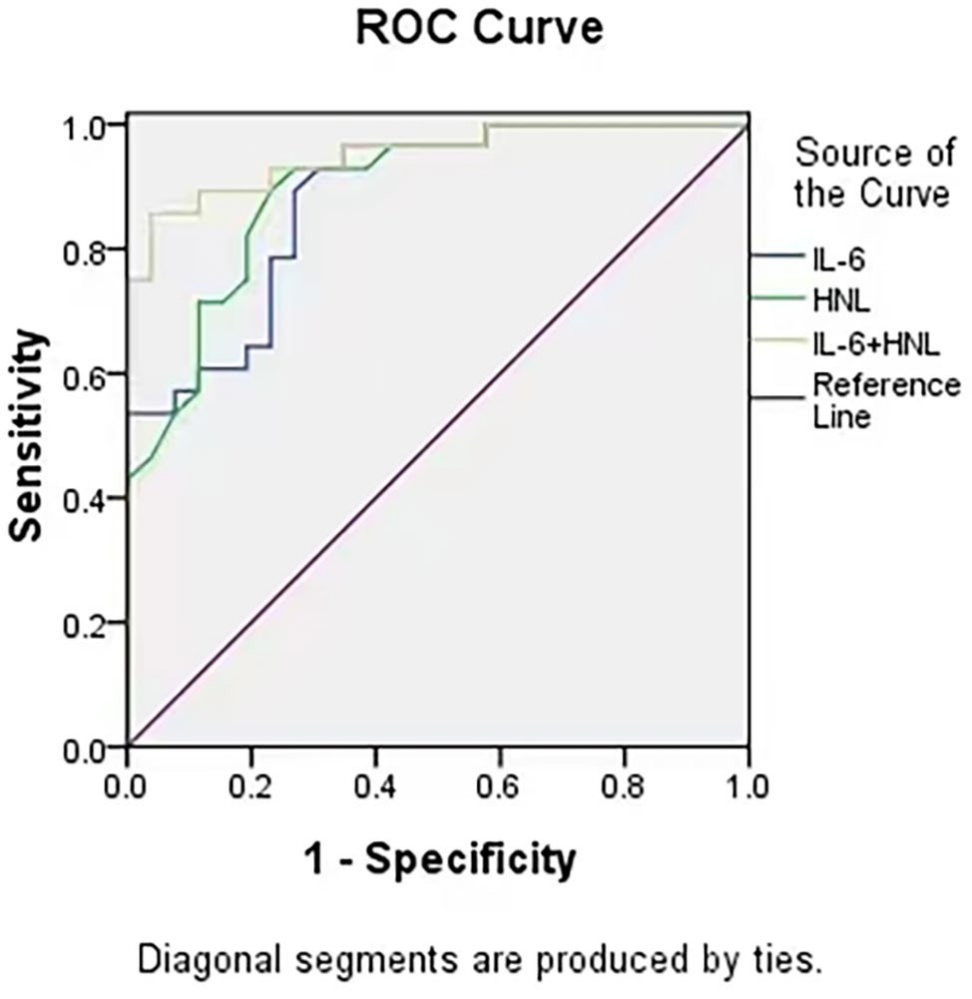
The ROC curve of SAP was predicted by IL-6 and HNL alone and in combination

According to the ROC curve analysis of IL-6, the area under the curve (AUC) was 0.881 (95% CI: 0.820 ~ 0.942), and the optimal cutoff value was 6.89 pg/mL with the sensitivity of 73.1% and specificity of 85.7%. As for the ROC curve analysis of HNL, the AUC was 0.896 (95% CI: 0.839 ~ 0.954), and the best cutoff value was 99.66ng/mL with the sensitivity of 76.9% and specificity of 89.3%. The AUC of the combination of IL-6 and HNL increased to 0.952 (95% CI: 0.914 ~ 0.989), and the sensitivity and specificity increased to 80.8% and 92.9%, respectively, with statistical significance (all *P* < 0.05), as shown in Table 4; Fig. 1.

**Table 4:** ROC curve analysis for prediction of SAP

	Optical cut-off	AUC	sensitivity%	Specificity %	SE	95%CI	*P* value
IL-6	6.89	0.881	73.1	85.7	0.031	0.820 ~ 0.942	<0.001
HNL	99.66	0.896	76.9	89.3	0.029	0.839 ~ 0.954	<0.001
IL-6 + HNL		0.952	80.8	92.9	0.019	0.914 ~ 0.989	<0.001

IL-6, interleukin-6; HNL, human neutrophil lipocalin

## Discussion

A total of 108 stroke patients were included in the study. we found that dysphagia, increased NIHSS scores, and increased IL-6 and HNL levelswere independent risk factors for stroke associated pneumonia. Furthermore, the levels of IL-6 ≥ 6.89 pg/mL and HNL ≥ 99.66ng/mL were considered as risk factors for AIS patients complicated with SAP. The indexes combined had higher predictive value for SAP.

After the onset of AIS, the ischemic nerve cells could activate the humanimmune system, stimulating the secretion of inflammatory cytokines, chemokines and other neurotoxic substances, leading to the damage of the blood-brain barrier and initiates a trainof inflammatory cascade reactions [14].Meanwhile, the inflammatory cells accessthe brain parenchyma viavascular endothelial cells, like polymorphonuclear neutrophils and lymphocytes, further mediating secondary damage of nerve cells and aggravating nerve function deficit. So as to alleviate the above damage, the body over-activates the sympathetic nervous system, parasympathetic nervous system and hypothalamic-pituitary-adrenal axis, resulting in post-stroke immunosuppression [15]. Consequently, neutrophils are stimulated and demarginated, and lymphocytes transform from proinflammatory type to anti-inflammatory type. The whole process could increase the susceptibility to infections such as SAP [16]. In this study, we found that IL-6 was in high secretionin pneumonia group than that in the non-pneumonia group. We hypothesize that the interaction between the clotting system and the inflammatory response plays a key role. First, coagulation is activated after ischemic stroke, which then leads to the release of pro-inflammatory cytokines and chemokines, resulting in inflammation. In addition, the inflammatory response can also induce coagulation activation through pro-inflammatory factors, the most common of which is IL-6, an important mediator that induces coagulation activation [17]. In addition,the elevation of IL-6 can induce the transcription of CRP gene, resulting in high expressionof CRP. Moreover, previous study indicatedthat pro-inflammatory cytokines,IL-6, could predict SAP [18].

In recent years, it has been found that HNL has potential diagnostic value in bacterial infection and has become a new and more advantageous biological marker [19, 20]. HNL is a protein secretedfrom the activated neutrophils, and when the immune system is stimulated and activated, HNL could be released into the extracellular surface. When the body is infected, the activation of neutrophils by pathogen couldincrease the expression ofHNL [21]. Therefore, HNL can be used as an indicator for the early diagnosis of acute bacterial infection, and its peak value is earlier than C-reactive protein, which is commonly used as an inflammatory indicator, and can also be used to monitor the efficacy, and it decreases 48 h after receiving effective treatment [22].

In this study, from the general clinical analysis, the proportion of dysphagia, NIHSS score, IL-6 and HNL levels in the group with pneumonia were higher than those in the group without pulmonary infectionsignificantly. The predominant site of pneumonia in all patients is the right middle and lower lobes of the lungs, which is believed to be associated with the anatomy of the right bronchial tree. The right main bronchus is relatively shorter and thicker, with a steep and direct course.Multivariate Logistic regression analysis demonstratedthat dysphagia and highNIHSS score were independent risk factors for SAP. The published study screenedseveralrisk factors of SAP, includingstroke severity (NIHSS > 15), swallowing disorder (Chinese version of Gugging Swallowing Screen < 15) and mechanical ventilation [23], which was consistent with our study. The elevated levels of IL-6 and HNL were also independent risk factors for SAP. IL-6 levels increase in the early stage of AIS patients, and when combined with infection, IL-6 and other pro-inflammatory cytokines can be further increased, thus further accelerating the inflammatory response procedure and leading to tissue and cell damage.Kwan suggested that IL-6 may be a key biomarker for predicting stroke associated infection and mortality in the first two years post stroke [24]. Studies have reportedthat the valueof HNL wascorrelatedto the number of activated neutrophils directly involved in inflammation. When acute bacterial infection occurs, the level of HNL in blood can be significantly increased within a few hours. Yang found that of combination of the National Institutes of Health Stroke Scale (A [2]DS [2])Score and IL-6 could significantly enhance the AUC efficacy of predicting SAP in patients with AIS inthe medical ward [25]. Therefore, the predictive value of IL-6 and HNL on SAP was analyzed separately and in combination. The ROC curve results showed that IL-6 ≥ 6.89pg/mL and HNL ≥ 99.66ng/mL were risk factors for AIS patients with SAP, both of which had good diagnostic value, and the area, sensitivity and specificity under the curve of combined detection of the two indicators were higher than that of single detection.

In conclusion, high levels of IL-6 and HNL could predict the likelihood of SAP events in patients with acute ischemic stroke,andthese testscan be quicklyand easily obtained, helping to select high-risk patients timelyand start intervention. Moreover, IL-6 ≥ 6.89 pg/mL and HNL ≥ 99.66ng/mL are risk factors for SAP in AIS patients, and combination detection of IL-6 and HNL has higher predictive value for SAP.

### Limitation

In this study, the sample size is relatively small and limited. So, the patient sample should be largerin the futurestudy. In addition, further large-scale studies are necessary forthe confirmation of the specific correlation between the IL-6, HNL and SAP in patients with AIS.

## Data Availability

The data that support the findings of this study may be available from the corresponding author (YC) upon reasonable request.

## Abbreviations

AIS: Acute Ischemic Stroke
AUC: Area Under The Curve
COPD: Chronic Obstructive Pulmonary Disease
ELISA: Enzymolytic Immunoassay
Hcy: Homocysteine
HNL: Human Neutrophil Lipocalin
IL-6: Interleukin-6
LDL-C: Low Density Lipoprotein Cholesterol
NIHSS: National Institutes Of Health Stroke Scale
P-PCT: Plasma Procalcitonin
ROC: Receiver Operating Characteristic
SAP: Stroke-Associated Pneumonia
TC: Total Cholesterol
